# Diphenhydramine Disrupts Sleep Architecture in 5XFAD Alzheimer’s Disease Model and Wild-Type Mice

**DOI:** 10.64898/2026.07.22.739929

**Published:** 2026-07-27

**Authors:** Margaret H. Copeland, Diane E. Youngstrom, Kathryn S. Konrad, Graham H. Diering, Ayland C. Letsinger, Leslie R. Aksu, Jerrel L. Yakel, Jesse D. Cushman

## Abstract

Sleep disruption is common in Alzheimer’s disease (AD). Diphenhydramine (DPH), a first-generation antihistamine with anticholinergic properties, is widely used as an over-the-counter sleep aid. We tested whether chronic DPH treatment alters sleep architecture in 5XFAD and wild-type mice. Female 5XFAD (n=16) and wild-type (WT) littermates (n=14) were implanted with wireless telemetry recording devices to measure electroencephalography (EEG), electromyography (EMG), temperature, and activity continuously. After a 24h baseline recording at 5 months of age, mice received oral DPH (10 mg/kg) or vehicle at ZT0 for one month. After this chronic treatment, sleep was recorded continuously for 48h during ongoing dosing. Sleep was scored as rapid eye movement (REM), non-rapid eye movement (NREM) 1, NREM2, or wake. A survival curve analysis was used to investigate the microarchitecture of sleep phases after chronic diphenhydramine treatment. At baseline, 5XFAD mice had more time in NREM1 than WT controls and had shorter REM and NREM2 bouts. Chronic DPH treatment fragmented NREM2 in both genotypes, reducing long NREM2 bouts. DPH increased total duration of NREM1 and REM during the active phase, which is analogous to daytime drowsiness in humans. DPH did not rescue 5XFAD sleep deficits; instead, DPH treatment exacerbated NREM2 fragmentation. Overall, chronic DPH use degrades sleep quality and increases fragmentation in both WT and AD-model mice, which questions the use of sedating anticholinergics as sleep aids, especially in AD.

## Full Text Availability

The license terms selected by the author(s) for this preprint version do not permit archiving in PMC. The full text is available from the preprint server.

